# Supplementary material for: Diabetes Does Not Increase the Risk of Hospitalization Due to COVID-19 in Patients Aged 50 Years or Older in Primary Care—APHOSDIAB—COVID-19 Multicenter Study
Source: J Clin Med. 2022 Apr 8;11(8):2092. doi: 10.3390/jcm11082092 (PMC9025638; doi:10.3390/jcm11082092)
Supplement: Supplementary file 1 [file jcm-11-02092-s001.zip › jcm-1631899-supplementary.pdf]

INVESTIGATORS

Researcher	Center	City
IMACULADA CANDELA GARCÍA	HEALTH CENTER SANTA POLA	SANTA POLA (ALICANTE)
FRANCISCO JAVIER ORTEGA RÍOS	HEALTH CENTER CAMPOS LAMPREANA	VILLARRÍN DE CAMPOS (ZAMORA)
JUAN FRANCISCO MERINO TORRES	HOSPITAL LA FE	VALENCIA
JOSEP MARIA VILASECA LLOBET	CAP COMTE BORRELL	BARCELONA
ROCÍO ORBEA GARCÍA	HEALTH CENTER CAMPOS LAMPREANA	VILLARRÍN DE CAMPOS (ZAMORA)
JAVIER DIEZ ESPINO	HEALTH CENTER SALUD TAFALLA	TAFALLA (NAVARRA)
JUAN MANUEL ZAZO MENARGUES	HEALTH CENTER EL RAVAL -ELX	ELCHE (ALICANTE)
ANDREA BERNABÉ CASANOVA	HEALTH CENTER EL RAVAL	ELCHE (ALICANTE)
JOSE LUIS PARDO FRANCO	HEALTH CENTER ORIHUELA I	ORIHUELA (ALICANTE)
JUAN MANUEL CALDERON GUADALUPE	HEALTH CENTER VILLARRIN DE CAMPOS	VILLARRIN DE CAMPO (ZAMORA)
LUIS AVILA LACHICA	HEALTH CENTER AXARQUIA OESTE	ALMACHAR
SANDRA ZARZA MARTÍNEZ	HEALTH CENTER LA PUEBLA DE ALMORADIEL	LA PUEBLA DE ALMORADIEL (TOLEDO)
SILVIA ZAMORA MESTRE	HEALTH CENTER ROGER DE FLOR	BARCELONA
MARIA BELEN BENITO BADORREY	HEALTH CENTER RAVAL SUD	BARCELONA
ENRIQUE CARRETERO ANIBARRO	HEALTH CENTER HERRERA	HERRERA (SEVILLA)
PILAR-COSIALES	HEALTH CENTER AZPILAGAÑA	PAMPLONA
SALVADOR PERTUSA MARTINEZ	HEALTH CENTER CABO HUERTAS	ALICANTE
IGNACIO PÁRRAGA	HEALTH CENTER ZONA VIII	ALBACETE
MANEL MATA CASES	HEALTH CENTER LA MINA	SANT ADRIÀ DE BESÒS (BARCELONA)
JOSE LUIS TORRES BAILE	HEALTH CENTER RODRIGUEZ PATERNA	LOGROÑO
ROSARIO IGLESIAS GONZÁLEZ	HEALTH CENTER PEDRO LAÍN ENTRALGO	ALCORCÓN (MADRID)
MARTA ROSET BARTROLI	HEALTH CENTER CONGRÉS	BARCELONA
BÁRBARA CÁNOVAS GAILLEMIN	TOLEDO HOSPITAL COMPLEX	TOLEDO
ANDER BURGAÑA AGOÜES	HEALTH CENTER SANT CUGAT	SANT CUGAT DEL VALLÈS (BARCELONA)
LAURA MOLA REYES	HOSPITAL CENTRAL DE LA DEFENSA GÓMEZ ULLA	MADRID
ISABEL SANTSALVADOR FONT	HEALTH CENTER VALLDOREIX	SANT CUGAT DEL VALLÈS (BARCELONA)
CRISTIAN MARCO ALACID	HOSPITAL VIRGEN DE LOS LIRIOS	ALCOY (ALICANTE)
CRISTINA TERCERO MACIÁ	ELCHE GENERAL HOSPITAL HEALTH CENTER SALUD EL RAVAL	ELCHE (ALICANTE)
GUAYENTE VERDES SANZ	ALCAÑIZ COUNTY HOSPITAL	ALCAÑIZ (TERUEL)
MARIA MERINO VIVEROS	GETAFE UNIVERSITY HOSPITAL	GETAFE (MADRID)
MARIA GUADALUPE GUIJARRO DE ARMAS	GETAFE UNIVERSITY HOSPITAL	GETAFE (MADRID)
CRISTINA NAVEA AGUILERA	GETAFE UNIVERSITY HOSPITAL	GETAFE (MADRID)
M ANGELES SALVADOR MILIAN	HEALTH CENTER CONGRES	BARCELONA

ELENA CARIDE MIANA	HEALTH CENTER LOS ÁNGELES	ALICANTE
ALBA MARTIN GONZALEZ	HOSPITAL 12th OCTOBER	MADRID
ROSA M MIRETE LOPEZ	HOSPITAL SAN JUAN DE ALICANTE	ALICANTE
MARIA JOSE BURCHES FELICIANO	ELCHE GENERAL UNIVERSITY HOSPITAL	ELCHE (ALICANTE)
NEREA AGUIRRE MORENO	UNIVERSITY HOSPITAL REY JUAN CARLOS	MÓSTOLES (MADRID)
LAURA MANJON MIGUELEZ	UNIVERSITY HOSPITAL CENTRAL DE ASTURIAS	OVIEDO
ANA CHICO	HOSPITAL SANTA CREU I SANT PAU	BARCELONA
CARLA MARISELA PACHECO URBINA	HOSPITAL MONTECELO	PONTEVEDRA
FRANCISCO JOSE POMARES GOMEZ	UNIVERSITY HOSPITAL SAN JUAN DE ALICANTE	SAN JUAN DE ALICANTE (ALICANTE)
LUÍS CUIXART COSTA	HEALTH CENTER ROGER DE FLOR.	BARCELONA
Mª ZELL DEL CASTILLO NESWEDA	HEALTH CENTER ROGER DE FLOR	BARCELONA
MARIA VICENTE SANTOS	HEALTH CENTER CAPUCHINOS	SALAMANCA
MONTSERRAT FREIXAS LAPORTA	HEALTH CENTER ROGER FLOR	BARCELONA
NATALIA LOPEZ PAREJA	HEALTH CENTER CONGRES	BARCELONA
ROSARIO IGLESIAS GONZÁLEZ	HEALTH CENTER PEDRO LAÍN ENTRALGO	ALCORCÓN (MADRID)
ANA Mª CEBRIÁN CUENCA	HEALTH CENTER CARTAGENA CASCO	CARTAGENA
MARIA TERESA MUR MARTÍ	HEALTH CENTER TERRASSA SUD	TERRASSA (BARCELONA)
VANESSA MARTÍNEZ AVILÉS	HEALTH CENTER RAVAL	ELCHE (ALICANTE)
SARA ARTOLA MENENDEZ	HEALTH CENTER JOSÉ MARVÁ	MADRID
ROSA MARIA MARTINEZ SAN CIPRIANO	HEALTH CENTER PLAZA DEL EJERCITO	VALLADOLID
MIREN ELIZARI RONCAL	HEALTH CENTER JOSÉ LUIS SANTAMARIA	BURGOS
FERNANDO ALVAREZ GUISASOLA	HEALTH CENTER RIBERA DEL ORBIGO	BENAVIDES DE ORBIGO (LEON)
CARLOS MIGUEL PETEIRO MIRANDA	HOSPITAL DE VILADECANS	VILADECANS (BARCELONA)
IGNACIO GARCIA HERRERO	HEALTH CENTER LOS BARREROS	CARTAGENA
EVA PERELLÓ CAMACHO	UNIVERSITY HOSPITAL SAN JUAN DE ALICANTE	ALICANTE
MACARENA LOPEZ VÁZQUEZ	BELLVITGE UNIVERSITY HOSPITAL	HOSPITALET DE LLOBREGAT (BARCELONA)
MARIA DELGADO SANDOVAL	UNIVERSITY HOSPITAL LA PAZ	MADRID
DIANA ROMERO GODOY	HEALTH CENTER MÁS FONT	VILADECANS (BARCELONA)

INVESTIGATORS

AGUIRRE MORENO, NEREA; HOSPITAL UNIVERSITARIO REY JUAN CARLOS; MÓSTOLES (MADRID); ALVAREZ GUISASOLA, FERNANDO; C.S.RIBERA DEL ORBIGO; BENAVIDES DE ORBIGO (LEON); ARTOLA MENENDEZ, SARA; Health Center JOSÉ MARVÁ; MADRID; AVILA LACHICA, LUIS;

C.S. AXARQUIA OESTE; ALMACHAR; BENITO BADORREY, MARIA BELEN; C. S. EL RAVAL; BARCELONA; BERNABÉ CASANOVA, ANDREA; C. S. EL RAVAL; ELCHE (ALICANTE); BURCHES FELICIANO, MARIA JOSE; GENERAL UNIVERSITY HOSPITAL DE ELCHE; ELCHE (ALICANTE); BURGAÑA AGOÜES, ANDER; C. S. SANT CUGAT; SANT CUGAT DEL VALLÈS (BARCELONA); CALDERON GUADALUPE, JUAN MANUEL; C.S. VILLARRIN DE CAMPOS; VILLARRIN DE CAMPO (ZAMORA); CANDELA GARCÍA, IMACULADA; C. S. SANTA POLA; SANTA POLA (ALICANTE); CÁNOVAS GAILLEMIN, BÁRBARA; COMPLEJO HOSPITALARIO DE TOLEDO; TOLEDO; CARIDE MIANA, ELENA; C. S. LOS ÁNGELES; ALICANTE; CARRETERO ANIBARRO . ENRIQUE; C. S. HERRERA; HERRERA (SEVILLA); CEBRIÁN CUENCA, ANA Mª; C. S. CARTAGENA CASCO; CARTAGENA; CHICO, ANA; HOSPITAL SANTA CREU I SANT PAU; BARCELONA; COSIALES, PILAR; C.S. AZPILAGAÑA; PAMPLONA; CUIXART COSTA, LUÍS; C. S. ROGER DE FLOR. ; BARCELONA; DEL CASTILLO NESWEDA, Mª ZELL; C. S. ROGER DE FLOR.; BARCELONA; DELGADO SANDOVAL, MARIA; HOSPITAL UNIVERSITARIO LA PAZ; MADRID; DIEZ ESPINO, JAVIER; C. S. SALUD TAFALLA; TAFALLA (NAVARRA); ELIZARI RONCAL, MIREN; C.S.JOSÉ LUIS SANTAMARIA; BURGOS; FREIXAS LAPORTA, MONTSERRAT; C. S. ROGER FLOR; BARCELONA; GARCIA HERRERO, IGNACIO; C. S. LOS BARREROS; CARTAGENA; GUIJARRO DE ARMAS DE ARMAS, MARIA GUADALUPE; HOSPITAL UNIVERSITARIO DE GETAFE; GETAFE (MADRID); IGLESIAS GONZÁLEZ, ROSARIO; C. S. PEDRO LAÍN ENTRALGO; ALCORCÓN (MADRID); IGLESIAS GONZÁLEZ, ROSARIO; C. S. PEDRO LAÍN ENTRALGO; ALCORCÓN (MADRID); LOPEZ PAREJA, NATALIA; C. S. CONGRES; BARCELONA; LOPEZ VÁZQUEZ, MACARENA; HOSPITAL UNIVERSITARIO DE BELLVITGE; HOSPITALET DE LLOBREGAT (BARCELONA); MANJON MIGUELEZ, LAURA; HOSPITAL UNIVERSITARIO CENTRAL DE ASTURIAS; OVIEDO; MARCO ALACID, CRISTIAN; HOSPITAL VIRGEN DE LOS LIRIOS; ALCOY (ALICANTE); MARTIN GONZALEZ, ALBA; HOSPITAL 12 DE OCTUBRE; MADRID; MARTINEZ SAN CIPRIANO, ROSA MARIA; C.S. PLAZA DEL EJERCITO; VALLADOLID; MARTÍNEZ AVILÉS, VANESSA; C. S. EL RAVAL; ELCHE (ALICANTE); MATA CASES, MANEL; C. S. LA MINA; SANT ADRIÀ DE BESÒS (BARCELONA); MERINO TORRES, JUAN FRANCISCO; HOSPITAL UNIVERSITARIO LA FE; VALENCIA; MERINO VIVEROS, MARIA; HOSPITAL UNIVERSITARIO DE GETAFE; GETAFE (MADRID); MIRETE LOPEZ, ROSA M; HOSPITAL UNIVERSITARIO SAN JUAN DE ALICANTE; ALICANTE; MOLA REYES, LAURA; HOSPITAL CENTRAL DE LA DEFENSA GÓMEZ ULLA; MADRID; MUR MARTÍ, MARIA TERESA; C. S. TERRASSA SUD; TERRASSA (BARCELONA); NAVEA AGUILERA, CRISTINA; HOSPITAL UNIVERSITARIO DE GETAFE; GETAFE (MADRID); ORBEA GARCÍA, ROCÍO; C.S. CAMPOS LAMPREANA; VILLARRÍN DE CAMPOS (ZAMORA); ORTEGA RÍOS, FRANCISCO JAVIER; C.S. CAMPOS LAMPREANA; VILLARRÍN DE CAMPOS (ZAMORA); PACHECO URBINA, CARLA MARISELA; HOSPITAL MONTECELO; PONTEVEDRA; PARDO FRANCO, JOSE LUIS; C. S. ORIHUELA I; ORIHUELA (ALICANTE); PÁRRAGA, IGNACIO; C. S. ZONA VIII; ALBACETE; PERELLÓ CAMACHO, EVA; HOSPITAL UNIVERSITARIO SAN JUAN DE ALICANTE; ALICANTE; PERTUSA MARTINEZ, SALVADOR; C. S. CABO HUERTAS; ALICANTE; PETEIRO MIRANDA, CARLOS MIGUEL;HOSPITAL DE VILADECANS; VILADECANS (BARCELONA); POMARES GOMEZ, FRANCISCO JOSE; HOSPITAL UNIVERSITARIO SAN JUAN DE ALICANTE; SAN JUAN DE ALICANTE (ALICANTE); ROMERO GODOY, DIANA; C. S. MÁS FONT; VILADECANS (BARCELONA); ROSET BARTROLI, MARTA; C. S. CONGRÉS; BARCELONA; SALVADOR MILIAN, M ANGELES; C. S. CONGRES; BARCELONA; SANTSALVADOR FONT, ISABEL; C. S. VALLDOREIX; SANT CUGAT DEL VALLÈS (BARCELONA); TERCERO MACIÁ, CRISTINA; GENERAL UNIVERSITY HOSPITAL DE ELCHE; ELCHE (ALICANTE); TORRES BAILE, JOSE LUIS; C. S. RODRIGUEZ PATERNA; LOGROÑO; VERDES SANZ, GUAYENTE; HOSPITAL COMARCAL DE ALCAÑIZ; ALCAÑIZ (TERUEL); VICENTE SANTOS, MARIA; C. S. CAPUCHINOS; SALAMANCA; VILASECA LLOBET, JOSEP MARIA; CAP COMTE BORRELL; BARCELONA; ZAMORA MESTRE, SILVIA; C. S. ROGER DE FLOR; BARCELONA; ZARZA MARTÍNEZ, SANDRA; C.S. LA PUEBLA DE ALMORADIEL; LA PUEBLA DE ALMORADIEL (TOLEDO); ZAZO MENARGUES, JUAN MANUEL;C. S. EL RAVAL; ELCHE (ALICANTE).
